# Atypical ^18^F-FDG PET-CT Findings in a Rare Case of Primary Hepatic Leiomyosarcoma

**DOI:** 10.3390/diagnostics14141502

**Published:** 2024-07-12

**Authors:** Miju Cheon, Hyunkyung Yi, Joo Young Ha, Min A Kim

**Affiliations:** 1 Department of Nuclear Medicine, Veterans Health Service Medical Center, Seoul 05368, Republic of Korea; 2 Division of Hematology and Oncology, Department of Internal Medicine, Veterans Health Service Medical Center, Seoul 05368, Republic of Korea; 3 Department of Pathology, Veterans Health Service Medical Center, Seoul 05368, Republic of Korea

**Keywords:** ^18^F-FDG, PET-CT, primary hepatic leiomyosarcoma, liver, leiomyosarcoma

## Abstract

The primary hepatic leiomyosarcoma is a rare malignant tumor arising from the smooth muscle cells in the hepatic vessels, bile ducts, and ligamentum teres. It is considered a subtype of hepatic sarcomas. We report awkward ^18^F-FDG PET-CT findings of a primary hepatic leiomyosarcoma masquerading as a benign hepatic tumor, which were confirmed by histopathological and immunohistochemical examinations in a 78-year-old woman.

A 78-year-old woman visited the emergency room after two weeks of persisting right flank and abdominal pain. Her past medical history included hypertension and hyperlipidemia. The patient had no other medical history, including previous liver diseases or alcohol abuse. The patient’s physical examination was unremarkable, and a laboratory analysis revealed a slightly high carcinoembryonic antigen (5.18 ng/mL). Other laboratory tests, including red bleed cell, white blood cell and platelet counts, liver, thyroid, and kidney functions, and hepatitis B surface antigen, hepatitis C virus antibody, α-fetoprotein, PIVKA-II, and carbohydrate 19-9, were normal.

To evaluate her right flank and abdominal pain, contrast-enhanced computed tomography (CT) scans were performed. They revealed two hypodense masses with a definite margin in hepatic segments IV and VII (Figure 1). The sizes of the two masses were 2.5 and 4 cm, respectively. The masses showed heterogeneous enhancement, but the contrast enhancement pattern was different from that of a hepatocellular carcinoma or cholangiocarcinoma. The findings were inconclusive and a metastatic hepatic tumor was regarded as the potential diagnosis.

^18^F-FDG positron emission tomography-CT (PET-CT) was conducted to determine the primary cancer origin and detect other metastatic sites. It revealed mild hypermetabolic low-density masses in the liver (Figure 2). The degrees of FDG metabolism of the hepatic masses were similar to that of other liver parenchyma. The SUVmax values of the hepatic masses were 4.58 and 3.92, respectively. The scan also revealed multiple hypermetabolic pulmonary nodules of various sizes in both lungs. In addition, it showed unexpected bone metastasis and findings of benign thyroid disease. However, no evidence of primary cancer from other organs was observed. The findings of the ^18^F-FDG PET-CT were challenging, and any rare type of hepatic malignant tumor was presented as a potential diagnosis. An abdominal CT showed that the hepatic masses were considered metastatic hepatic tumors, and no primary cancer was evident, and a chest CT only showed findings of pulmonary metastasis. However, in the ^18^F-FDG PET-CT, the liver lesion cannot be considered as a metastatic hepatic tumor because only a slight increase in FDG metabolism is barely distinguishable from the surrounding liver parenchyma, and the margin is relatively clear. Therefore, it is considered a benign hepatic tumor or a rare type of primary hepatic tumor, rather than a metastatic hepatic tumor or hepatocellular carcinoma. So, we performed a biopsy on the hepatic mass.

An ultrasonography biopsy subsequently revealed atypical cells, a cellular tumor with smooth muscle origin, and a high-proliferation tumor (Figure 3). These findings were consistent with a diagnosis of leiomyosarcoma. No other primary malignancy was detected via other examinations, such as gastrointestinal endoscopy, CT, and PET-CT, so the lesions were considered as a primary hepatic leiomyosarcoma (PHL).

PHL is an extremely rare malignancy with an estimated 0.2% prevalence [1,2,3]. The underlying pathogenic mechanisms have not been identified yet. There were no remarkable serological markers or clinical symptoms for PHL. In addition, the imaging findings were very diverse, including single or multiple lesions, hypervascular lesions, and hypovascular lesions. These non-specific findings might lead to a delayed diagnosis. Due to the rareness of the disease, the standard treatment has yet to be well defined. However, surgical resection followed by adjuvant chemotherapy is being used widely. The effectiveness of adjuvant chemotherapy or radiotherapy, liver transplantation, or transarterial chemoembolization remains debated. PHL is usually diagnosed at an advanced stage because its presenting symptoms are generally vague and non-specific. An awareness of the disease and an early diagnosis are essential due to its delayed diagnosis and aggressive behavior.

The reports of primary hepatic leiomyosarcoma in the English literature were also rare. Moreover, there are only three with ^18^F-FDG PET-CT findings, and all cases showed a marked increase in FDG metabolism with an SUVmax of eight or more [4,5,6]. However, in the present case, the mass showed an only mildly increased FDG metabolism similar to other normal liver parenchyma. To the best of our knowledge, no previous case reports show a mildly increased FDG metabolism of a primary hepatic leiomyosarcoma. In addition, all previously reported ^18^F-FDG PET-CT findings of leiomyosarcomas originating from the various organs such as the liver, uterus, oral cavity, adrenal gland, urinary bladder, breast, orbit, renal, and mediastinal also showed a markedly increased FDG metabolism [7,8,9,10,11,12,13,14,15,16,17], but this case was different. Unlike previous reports, this case showed a mild increase in FDG metabolism in leiomyosarcoma lesions. However, the exact cause was unknown. Depending on the degree of differentiation or necrosis, different degrees of FDG metabolism might be seen, but it is not clear. Therefore, collecting and analyzing several cases in the future will be necessary. According to previous reports on the role of the ^18^F-FDG PET-CT in leiomyosarcomas originating from various organs, the higher the value of metabolic parameters such as SUVmax in the ^18^F-FDG PET-CT, the worse the prognosis. It also might help diagnose and stage leiomyosarcoma [18,19,20]. However, the incidence of leiomyosarcoma is not high, so further high-quality studies are still needed on the role of the ^18^F-FDG PET-CT in outcomes in leiomyosarcoma patients. In conclusion, the primary hepatic leiomyosarcoma is rare and difficult to diagnose before histological confirmation. Unlike previous reports, this case confirmed that a primary hepatic leiomyosarcoma did not necessarily show a marked increased FDG metabolism in the ^18^F-FDG PET-CT. Further studies are needed to elucidate and better understand this rare disease.

## Figures and Tables

**Figure 1:**
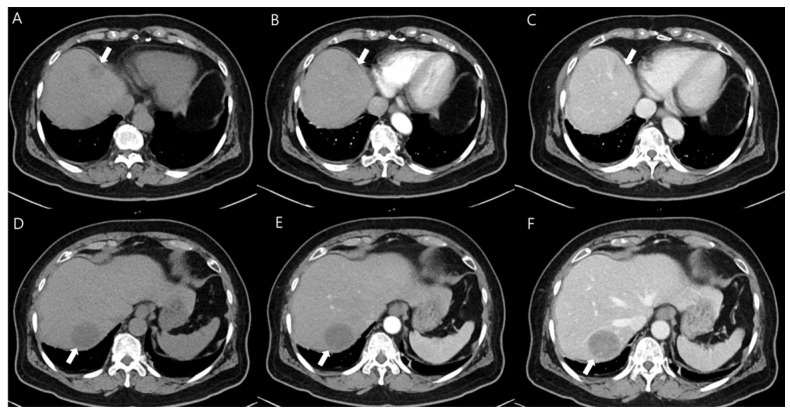
Contrast-enhanced abdominal CT images showed two low-density masses (white arrows) in hepatic segments IV and VII (**A**,**D**). In the arterial phase, the masses show no evident enhancement (**B**,**E**). In the venous phase, slightly heterogeneous enhancing masses were noted (**C**,**F**).

**Figure 2:**
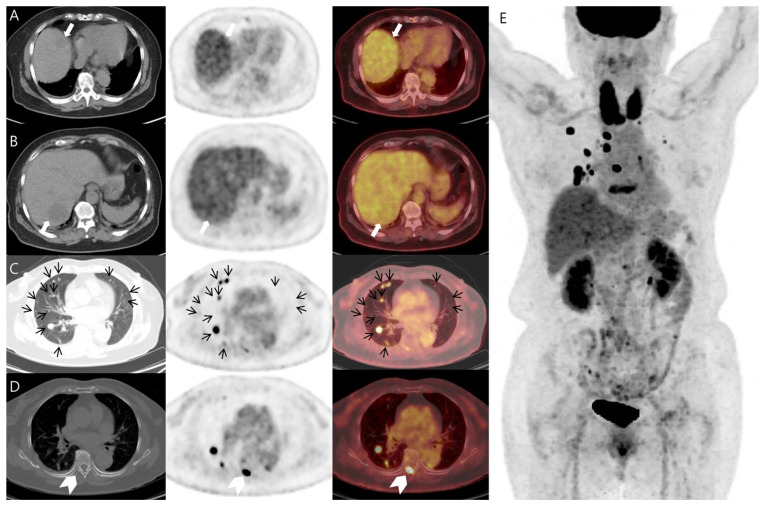
Transaxial images (**A**–**D**) and three-dimensional maximum intensity projection (**E**) of the ^18^F-FDG PET-CT, which demonstrated mild hypermetabolic low-density mass in the liver (white arrows), multiple hypermetabolic pulmonary nodules (black arrows), and a hypermetabolic bone lesion in the thoracic spine (white arrowhead). There was also marked and diffuse increased FDG metabolism in the thyroid gland, which represents thyroiditis.

**Figure 3:**
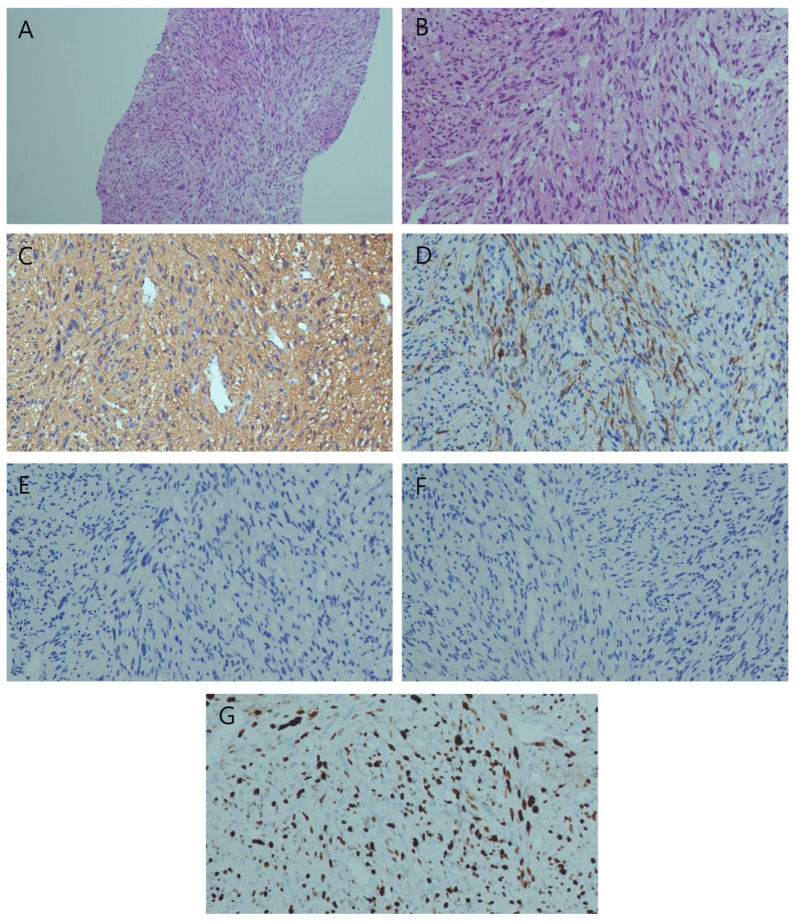
A microscopic examination showed a cellular tumor with a fascicular pattern ((**A**), H&E, ×100), and the tumor cells had atypism and mitosis ((**B**), H&E, ×200). Immunohistochemical staining revealed that the tumor cells were positive for smooth muscle actin ((**C**), ×200) and Desmin ((**D**), ×200). It also showed a high expression for Ki-67 ((**G**), ×200). In contrast, the tumor cells were negative for CK ((**E**), ×200), Hepatocyte ((**F**), ×200), CD34, CD117, S-100, TTF-1, human melanoma black 45 (HMB45), and Melan A.

## Data Availability

The data that support the findings of this study are available from the corresponding author, M.C., upon reasonable request.
